# Viral delivery of abnormal tau into subiculum projecting entorhinal cells impairs temporal‐association memory in a mouse model of early Alzheimer's Disease

**DOI:** 10.1002/alz70855_098294

**Published:** 2025-12-23

**Authors:** James Eric Carmichael, Martha Liu, TianMeng Xu, Pola S Tuduri, Michelle Ding, Frédéric S Manseau, Sylvain Williams

**Affiliations:** ^1^ Douglas Research Centre, Montreal, QC, Canada; ^2^ McGill University, Montreal, QC, Canada; ^3^ UC San Diego, La Jolla, CA, USA

## Abstract

**Background:**

Among the earliest signs of preclinical Alzheimer's disease (AD) are navigation impairments and the presence of abnormal hyperphosphorylated tau (aTau) proteins in the entorhinal cortex (EC), a brain region that encodes space and movement. As AD progresses and memory impairments develop, EC aTau spreads to the subiculum (Sub), a key region for memory encoding and recall. To better understand the impact of aTau progression from EC to Sub on neurophysiology and memory we designed a mouse model that seeds human mutant tau in Sub projecting EC neurons.

**Method:**

To best approximate the spread of aTau in early AD, we injected a cre‐dependent virus that selectively deposits mutant human tau (AAV2/9‐Flex‐P301L‐GFP) in a transgenic mouse line that limits cre expression to EC layers 2/3 cells (tg(Oxr‐1cre)C14Stl/J). By injecting into Sub, only cre+ cells projecting to Sub are initially transfected by the virus. Male and female mice were given between 4 weeks to 12 months for aTau to express. Mice were then tested on spatial memory (water maze), temporal‐association & contextual memory (trace‐fear conditioning), and anxiety (elevated plus maze) tasks. Task performance was compared to cre ‐/‐ littermates who received the same virus injections but did not express the tau virus.

**Results:**

Our viral strategy effectively limited transfection to Sub projecting EC layer 2/3 neurons. Immunofluorescence imaging confirmed that both human tau (AT7) and phosphorylated tau (AT8) were present in the EC‐Sub circuit as early as 4 weeks post injection. Mice with hyperphosphorylated tau in Sub projecting EC neurons showed impaired trace memory (*p* = 0.027) while spatial and contextual memory remained intact. There were no differences in anxiety levels.

**Conclusion:**

We have shown that selective delivery of human mutant tau to the EC‐Sub circuit impairs temporal‐association memory. These data are in line with previous reports of circuit inactivations disrupting association memory, and offer a new model to probe specific memory loss and guide interventions to slow aTau spread and rescue memory.

